# Forecasting Stock Price Trends by Analyzing Economic Reports With Analyst Profiles

**DOI:** 10.3389/frai.2022.866723

**Published:** 2022-06-07

**Authors:** Masahiro Suzuki, Hiroki Sakaji, Kiyoshi Izumi, Yasushi Ishikawa

**Affiliations:** ^1^Department of Systems Innovation, School of Engineering, The University of Tokyo, Tokyo, Japan; ^2^Nikko Asset Management Co., Ltd., Tokyo, Japan

**Keywords:** financial report, text mining, profile, BERT, stock prediction, net income

## Abstract

This article proposes a methodology to forecast the movements of analysts' estimated net income and stock prices using analyst profiles. Our methodology is based on applying natural language processing and neural networks in the context of analyst reports. First, we apply the proposed method to extract opinion sentences from the analyst report while classifying the remaining parts as non-opinion sentences. Then, we employ the proposed method to forecast the movements of analysts' estimated net income and stock price by inputting the opinion and non-opinion sentences into separate neural networks. In addition to analyst reports, we input analyst profiles to the networks. As analyst profiles, we used the name of an analyst, the securities company to which the analyst belongs, the sector which the analyst covers, and the analyst ranking. Consequently, we obtain an indication that the analyst profile effectively improves the model forecasts. However, classifying analyst reports into opinion and non-opinion sentences is insignificant for the forecasts.

## 1. Introduction

Investors need to search for market information such as stock prices and fundamental information such as companies' sales, earnings, and business conditions. A search engine can provide a variety of information about a company. A company's website also provides investor relation (IR) information on its business performance and financial information. A newspaper or a news site provides stock price charts and new information about companies. The trends of individual investors can be seen on social networking sites and message boards. However, IR information and news articles are limited to factual information, and social networking services (SNS) and message boards are not highly reliable. It is not easy to gather useful information for investment decisions. In this situation, analyst reports have been attracting attention. Analyst reports are written by analysts affiliated with securities companies that evaluate each stock, taking into account earnings guidance, valuation of stock price, press releases, macroeconomy trends, etc. Analyst report includes facts, such as financial results, stock prices, and information announced by the company. It also includes analysts' forecasts of future earnings and stock price performance and objective information which analysts think is worth including. Although analyst report contains useful information and unique information, it is difficult for investors, who manage many stocks, to read all analyst reports because many reports are issued during a period of the earnings announcements. Thus, Several studies applied text mining to analyst reports. Huang et al. (2014) applied Naive Bayes to words extracted from analyst reports to analyze the market's reaction to analyst reports. Suzuki et al. (2020) input the text of analyst reports into long short-term memory (LSTM) to predict stock price movements.

Although the text of an analyst report is used, the information of an analyst who writes text and evaluates stocks is not considered. For large-cap stocks, analyst reports are issued by several analysts, and it is essential to know which analyst writes the report. Some previous studies focused on the text. Amir et al. (2016) constructed user-specific distributed representations from past postings of users to detect sarcasm in text posted on Twitter. They showed that inputting distributed representations in addition to texts improves detection accuracy. Wang et al. (2019) proposed a method to construct representations of users and topics from financial postings on Weibo. They applied the representations to sentiment analysis and showed that the accuracy was improved compared to the baseline. They are indexed and characterized from the textual information; however, information other than the original texts is not utilized.

**Figure 1:**
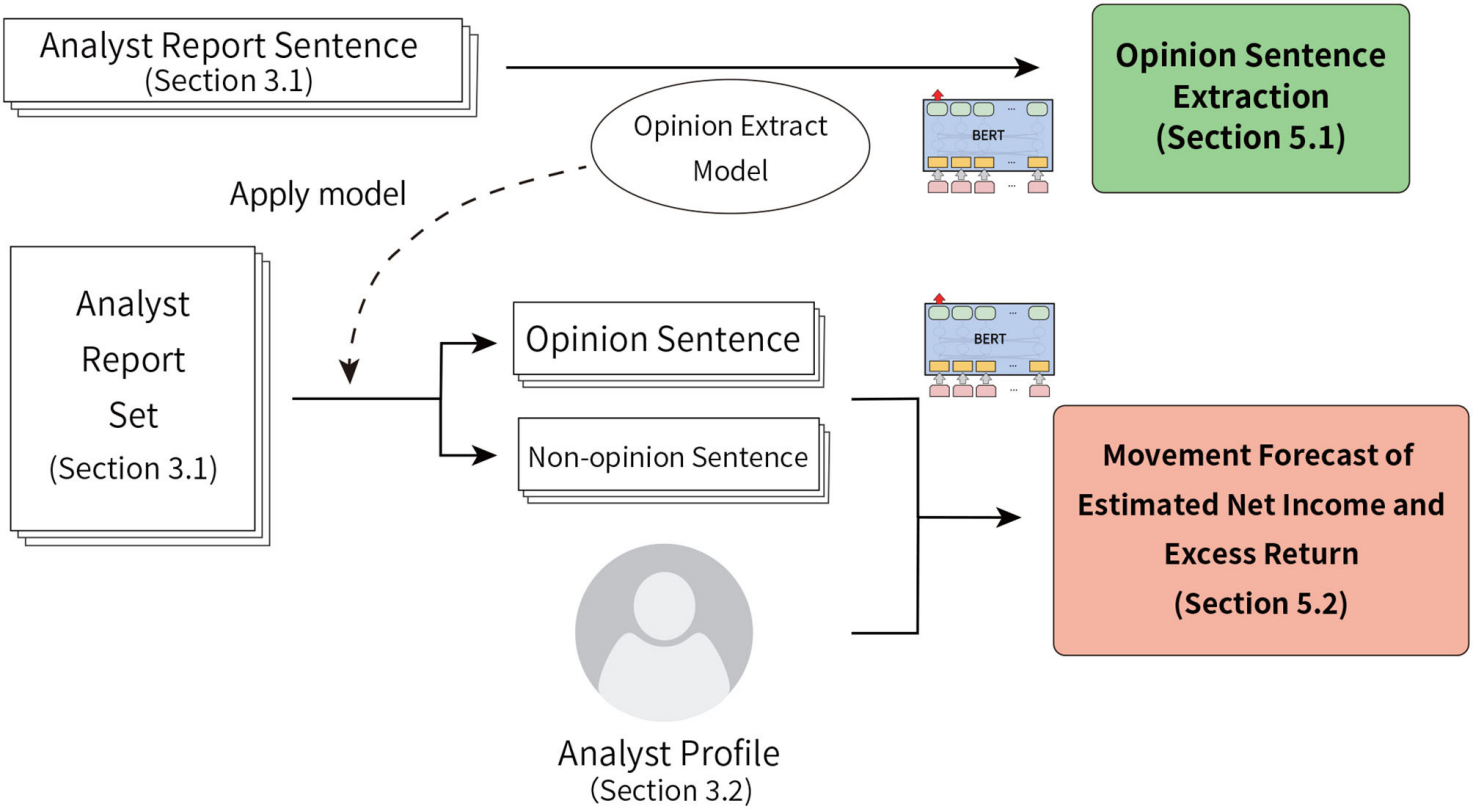
Overview of this research. In this study, we conduct two experiments: opinion sentence extraction and the movement forecasts of analysts' estimated net income and excess return.

Figure 1 shows the overview of this research. In this study, with the aim to forecast excess return of stock prices and the change rate of analysts' estimated net income, we propose a method to utilize unique information from the analyst reports using opinion and non-opinion sentences extracted from analyst reports and analyst's profiles. In addition to the main texts of analyst reports, the information (profile) of the analyst (who is the author) is used for input, and the effectiveness is confirmed. We use four types of information below as the analyst profile.

- Analyst name
- Securities company to which an analyst belongs
- Industries in which an analyst is in charge of
- Analyst ranking

The securities company for which an analyst works is also used as one of the analyst profile information.

## 2. Related Study

Several studies have been conducted on financial text mining to forecast financial markets. Regarding the research using financial news, Wuthrich et al. (1998) predicted daily movements of five indices using news articles published on the Internet. They constructed the rule to predict by combining news articles, index values, and some keywords. They found that textual information with bag-of-words in addition to numeric time-series data increases the quality of the input. Low et al. (2001) proposed a semantic expectation-based knowledge extraction methodology for extracting causal relations using WordNet as a thesaurus to extract terms that represent movement concepts. Koppel and Shtrimberg (2006) proposed a method for classifying the news stories of a company according to their apparent impacts on the performance of the company's stock price. Tetlock et al. (2008) showed that negative words in reports of financial institutions predict the low profits and the market incorporates the information in negative words with a slight delay. Although negative words related to fundamentals are particularly useful in predicting earnings and returns, they showed that the market underestimates the information. Bar-Haim et al. (2011) proposed a framework to identify expert investors and used it to predict stock price rise from stock tweets using a support vector machine (SVM) classifier. They trained the classifier that directly learned the relationship between the content of a tweet and stock prices. The user who was writing tweets that could discriminate the rise/fall of the stock price was learned as a specialist. Consequently, they constructed the classifier trained only by the set of tweets of the identified experts.

Regarding the research using the documents published by financial institutions and companies, Milea et al. (2010) predicted the Morgan Stanley Capital International (MSCI) euro index (upwards, downwards, or constant) based on fuzzy grammar fragments extracted from a report published by the European Central Bank. Brown and Tucker (2011) showed that the market price reaction weakens despite the increase in disclosed information, and the usefulness of management's discussion and analysis (MD&A) has declined. They also showed that analysts do not revise their earnings forecasts and equity investors are more responsive to the disclosure of MD&A. Loughran and Mcdonald (2014) showed that the text file size is more effective as an explanatory variable in the readability of the disclosure documents of financial status than Fog Index, which is one of the readability indicators; it is calculated from the number of words per sentence and the ratio of words with three or more syllables. They obtained that the larger the file size of a 10-K document, the greater the volatility of abnormal returns after disclosure and the greater the variance of analyst expectations by regression analysis.

Regarding the research using the texts on financial SNS, Bollen et al. (2011) showed that mood states obtained from tweets are relevant for forecasting Dow Jones Industrial Average (DJIA). They applied Opinion Finder and Google-Profile of Mood States (G-POMS), a mood analysis tool, to extract six types of mood from tweets: Calm, Alert, Sure, Vital, Kind, and Happy. They also used self-organizing fuzzy neural networks to predict rises and drops with an accuracy of more than 80%. They found that mood states in terms of positive or negative mood are ineffective in forecasting; meanwhile, those labeled “Calm” are effective. Chen et al. (2014) showed that opinions posted on popular social media with investors strongly predict future stock returns and market surprise reactions to earnings.

Regarding the study of applying sentiment to financial documents, Schumaker and Chen (2009) proposed a machine learning method to forecast stock prices by analyzing financial news articles. Their model forecasted indicators and stock prices. Schumaker et al. (2012) united their approach using sentiment analysis. The estimated stock prices after releasing financial news articles using SVM. Ito et al. proposed a neural network model for visualizing online financial textual data (Ito et al., 2018a,b). Their proposed model acquired word sentiment and its category. Guijarro et al. (2019) analyzed the impact of investors' moods on market liquidity. They conducted a sentiment analysis of tweets related to the S&P 500 Index. Vu et al. (2012) proposed a method using a decision tree classifier to predict the daily price movements of four famous tech stocks. They applied sentiment analysis, semantic orientation, and movements of previous days as features for tweets. They obtained an accuracy of more than 75%. Oliveira et al. (2017) constructed sentiment and attention indicators extracted from microblogs and used machine learning-based methods for financial tweets sentiment classification of predicting daily stock market variables. They tested five machine learning-based methods for financial tweets sentiment classification using the indicators. Ranco et al. (2015) analyzed the effects of sentiments of tweets about companies on DJIA 30 prices using SVM. They found a dependence between stock price returns and Twitter sentiments. Smailović et al. (2013) showed causality between sentiment polarity of tweets and daily return of closing prices. They used sentiment derived from an SVM model to classify the tweets into positive, negative, and neutral categories.

Regarding financial text mining for the Japanese language, Sakaji et al. (2008) proposed a method to automatically extract basis expressions that indicate economic trends from newspaper articles using a statistical method. Additionally, Sakaji et al. (2017) proposed an unsupervised approach to discover rare causal knowledge from financial statement summaries. Their method extracted basis expressions and causal knowledge using syntactic patterns. Kitamori et al. (2017) proposed a method for extracting and classifying sentences indicating business performance forecasts and economic forecasts from summaries of financial statements. This classification method was based on a neural network using a semi-supervised approach.

Regarding a study using analyst reports, Asquith et al. (2005) showed that the revision of the target stock price in an analyst report has a significant positive correlation with the market reaction. They also showed that half of the analyst reports contained new information in addition to previously known information. They obtained that the market is most responsive to rating downgrades in analyst reports. Huang et al. (2014) showed that analyst reports play two roles, such as information interpretation in conference calls and information discovery not mentioned in conference calls, using the topic model (LDA).

In this research, we apply the network combining bidirectional encoder representations from transformers (BERT) (Devlin et al., 2019) and multilayer perceptron (MLP). BERT is a language model that uses Transformer (Vaswani et al., 2017), which achieves high performance without using a complicated structure, such as LSTM, in bidirectional ways. BERT achieved higher accuracy than conventional methods, such as LSTM, by performing pre-training using a large corpus. In financial text mining, there are various studies applying BERT. Araci (2019) applied pre-trained BERT for two financial sentiment analysis datasets. Araci showed that the BERT model achieves a higher F1 score than other models such as LSTM. Del Corro and Hoffart (2021) proposed a method to automatically identify financially relevant news by applying BERT. They showed that the method ranks relevant news highly and positively correlated with the accuracy of the initial stock price prediction task. Kim and Yoon (2021) proposed a model to predict impending bankruptcies using BERT. They showed that BERT outperforms dictionary-based predictions and Word2Vec-based predictions. Taguchi et al. (2021) showed analysts' sentiment toward individual stocks is useful in predicting the macroeconomic index. They applied BERT to create polarity indexes from analyst reports.

## 3. Data

### 3.1. Analyst Report

We use two types of analyst reports: analyst report set and analyst report sentences. The analyst report set consists of 75,440 analyst reports published from January 2016 to September 2020 by Japanese securities companies. The analyst report sentences consist of 2,204 sentences extracted from 100 analyst reports which are randomly extracted from 10,100 analyst reports published in 2017. These sentences are used for the training data of the experiment of opinion sentence extraction. In this study, an opinion sentence is defined as one expressing an analyst's subjective views, such as ratings for future stock prices, sales or forecasted net earnings for the next year, and backgrounds of current sales. A non-opinion sentence is defined as one expressing objective facts such as financial results. Table 1 presents examples of opinion and non-opinion sentences. After manual tagging by three of the authors, including a fund manager who uses the analyst report for operation, 1,180 sentences are labeled as opinion sentences, whereas the remaining 1,024 sentences are labeled as non-opinion sentences. Tagging was done by each of the three people, and the final decision was made by the fund manager.

**Table 1:** Typical examples of opinion and non-opinion sentences in analyst reports. English follows Japanese.

**Type**	**Sentence**
Opinion	2Q実績を踏まえ,業績予想を下方修正する
	We will revise our earnings forecast downwards based on 2Q results.
Opinion	収益性低下の要因として考えられるのは,新たな生産拠点 の立ち上げや研究開発投資である
	The factors that could reduce profitability are the launch of new production bases and R&D investment.
Non-opinion	今期の売り上げは100億円と,過去最高になった
	This term sales reached a record high of 10 billion yen.
Non-opinion	配当は19年9月中間期120円,期末150円の合計270円を 予定している
	The dividend is planned to be 270 yen for the interim period of September 2007, 120 yen and 150 yen at the end of the year.

The sentences contained in these analyst reports are classified into opinion and non-opinion sentences using the model constructed in Section 5.1. They are used for the forecast experiments of analysts' estimated net income and excess return. Among the 1,681,114 sentences in the reports, 9,02,652 and 7,78,462 sentences are classified as an opinion and non-opinion sentences, respectively.

### 3.2. Analyst Profile

In this study, the analysts' information, who are the authors of analyst reports, is used as the text information of analyst reports. The name of an analyst, an affiliated securities company, industries in charge, and an analyst ranking are used as the analyst profile. The analyst's name is used to indicate which analyst writes the analyst report. From the analyst report set, we extract 345 analysts. All the analysts belong to one of the five securities companies that publish the analyst reports used in this research. We use two types of industries: Tokyo Stock Exchange (TSE) 33 industries and those mapped to 10 industries. The analyst ranking uses the latest information of the first to fifth analyst rankings by Nikkei Veritas as of the publication date of the analyst report. In this research, we use all the information available that accompanies the analyst report. Though other information such as years of experience as an analyst might be, this information is not publicly available and is difficult to collect. Therefore, the information mentioned above is used in this research.

### 3.3. Data Used for the Forecast of the Analyst's Estimated Net Income

This study uses the rate of change in analyst's estimated net income to forecast the rise and fall of the net income. First, the analyst's estimated net income is calculated. Let NI(*t*) be the analyst's estimated net income of the target stock for the last 12 months at a point *t*. NI(*t*) is calculated by prorating the estimated net income for the current term and that for the next term in the latest report at the time *t*. Proration is used because using only the current term estimated net income or the next term estimated net income makes a jump when crossing the fiscal year-end and the target period of the estimated net income will not be constant. Consider an example of 31 December 2021. For many companies with March settlements, an analyst estimates net income for the current term from April 2021 to March 2022 and that for the next term from April 2022 to March 2023. Therefore, the estimated net income for the next 12 months from 31 December 2021, is calculated as proration of the estimated net income for the three months (from January 1 to March 31) of the current term until March 2022 and the estimated net income for the nine months (from April 1 to December 31) of the next term until March 2023. Let NI′, NI^′′^ be the current term estimated net income and the next term estimated net income. Then NI(*t*) is calculated as


(1) $$\begin{array}{l} \text{NI}\left(t\right)=\text{N}{\text{I}}^{\prime }\times \frac{3}{12}+\text{N}{\text{I}}^{\prime \prime }\times \frac{9}{12}. \end{array}$$

Let the change rate of the analyst's estimated net income for 60 business days (about three months) from the day after the analyst report is published be FR(60). We apply the term of 60 business days (about three months) because analyst reports are usually published quarterly (approximately every three months) in line with quarterly financial settlements. Then, it is calculated as


(2) $$\begin{array}{l} \text{FR}\left(60\right)=\{\begin{array}{ll} \frac{\text{NI}\left(t+60\right)-\text{NI}\left(t\right)}{\left|\text{NI}\left(t\right)\right|} & \left(\text{NI}\left(t\right)\neq 0\right)\\ sgn\left(\text{NI}\left(t+60\right)-\text{NI}\left(t\right)\right) & \left(\text{NI}\left(t\right)=0\right). \end{array} \end{array}$$

Here, sgn(·) is a sign function, which returns 1 when the input is positive, 0 when the input is 0, and –1 when the input is negative. In the experiment, binary classification is conducted according to the change rate of the analyst's estimated net income FR(60). The median value of the training data is used as the threshold value of FR(60).

### 3.4. Data Used for Forecast of Stock Price Movement

In the stock price movement forecast, the excess returns, which are the returns of stocks exceeding the benchmark, are forecasted as positive or negative. Therefore, the stock prices of stocks and the benchmark Tokyo Stock Price Index (TOPIX) are used. Let the stock price and TOPIX on the day after the analyst report issued date be *C*_0_ and *T*_0_, respectively. An analyst report is published after the end of the trading day to avoid an impact on the market. Then, the information from the analyst report is incorporated into the market the day after the issue date. Therefore, the value on the day after the issue date is used in this study. Similarly, let those 60 business days (about three months) after the issue date be *C*_60_ and *T*_60_, respectively. The closing price is used for each index. Let the excess return after 60 business days (about three months) be ER(60), which is calculated as


(3) $$\begin{array}{l} \text{ER}\left(60\right)=\frac{\left(C_{60}-C_{0}\right)}{C_{0}}-\frac{\left(T_{60}-T_{0}\right)}{T_{0}}. \end{array}$$

For institutional investors, who are relatively valued compared to benchmarks such as TOPIX, predictability of excess returns is more important than that of simple returns. For this reason, the excess return is used. We classify excess returns into positive and negative values for each analyst report. Among the 75,440 reports, 36,163 and 39,277 were classified as positive and negative excess returns, respectively.

## 4. Method

### 4.1. Opinion Sentence Extraction

Bidirectional Encoder Representations from Transformers (BERT) (Devlin et al., 2019) is used to construct the opinion sentence extraction model. Natural language models such as BERT apply unsupervised learning to a large number of documents for pre-training. Pre-training of BERT consists of the masked language model and the next sentence prediction. In the masked language model, the model predicts a masked word. However, in the next sentence prediction, the model predicts whether two sentences are continuous or not. These tasks make it possible to capture the meaning of words and phrases and the structure of sentences. Using a pre-trained model constructed by a large corpus and fine-tuning with target data, the model can be more effective than one training with the target data from scratch. This strategy has resulted in significant performance improvements in natural language processing tasks. The pre-trained Japanese BERT model by Tohoku University ^1^.

### 4.2. Forecasting Movements of Net Income and Excess Return

**Figure 2:**
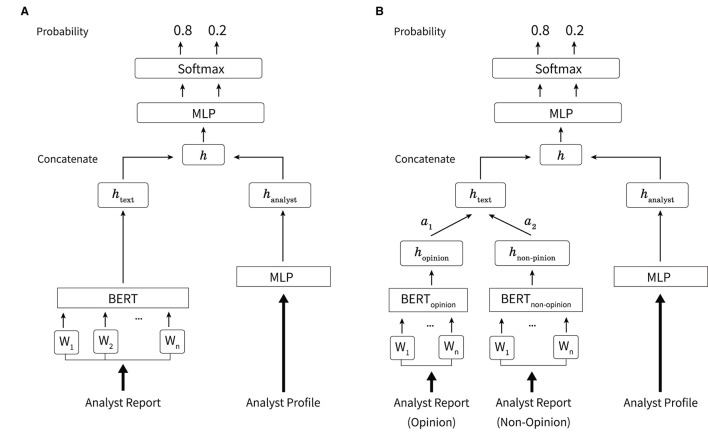
Overview of the method used in this study. **(A)** The body text of the analyst report is processed using BERT and output as *h*_text_. The analyst profile is processed using MLP and output as *h*_analyst_. Here, *h* is obtained from the concatenation of *h*_text_ and *h*_analyst_. MLP and softmax functions are applied to output the probability of each label. **(B)** Overview of the case where the opinion and non-opinion sentences in an analyst report are input separately. The opinion and non-opinion sentences are processed using BERT and output as *h*_opinion_, *h*_non−opinion_, respectively. These are weighted by the weights of attention mechanisms such as α_1_, α_2_, respectively, and *h*_text_ is obtained from the sum.

**Figure 3:**
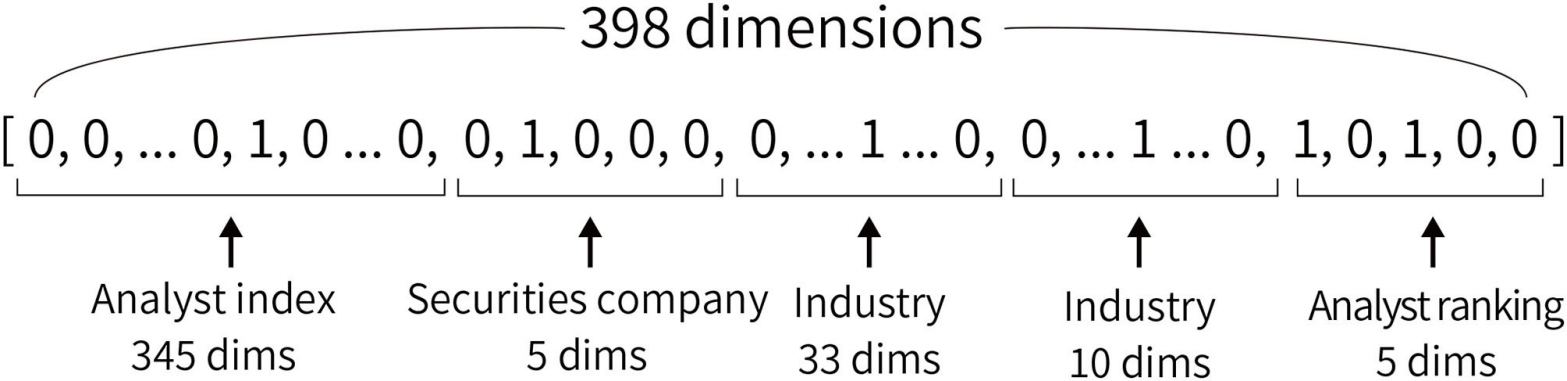
An example of a vector constructed using an analyst profile. All elements are composed of 0 or 1. In the analyst index, one of the 345 dimensions is 1, and the others are 0. We use two types of industries: TSE 33 industries and those mapped to 10 industries.

Figure 2A shows the overview of the network used in this study. In forecasting movements of net income and stock price, the text of analyst reports and profiles are input. The model outputs the rate of change in a two-class classification of estimated net income and excess returns. The name, affiliated securities company, industry in charge, and the analyst ranking described in Section 3.2 are used as the analyst profile. For each of this information, we create a vector for each analyst report, shown in Figure 3, where the corresponding parts are 1 and the other parts are 0. For the names of analysts, indexes are given to all 345 analysts who are the authors of the analyst reports. Then we construct 345-dimensional vectors with 1 for the index of the analyst and 0 for the others. We also construct five-dimensional vectors to indicate the securities companies since this study uses analyst reports issued by five securities companies. Vectors with 345 dimensions for the name, five dimensions for the affiliated securities company, 33 and ten dimensions for the industry in charge, and five dimensions for the analyst ranking are constructed. A total of 398 dimension vectors is used as the analyst profile.

Let the analyst report and profile of the author of the report be *x*_text_ and *x*_analyst_, respectively. Then, *h*_text_ is obtained by inputting *x*_text_ to BERT as


(4) $$\begin{array}{l} h_{\text{text}}=\text{BERT}\left(x_{\text{text}}\right). \end{array}$$

Here, BERT(·) is referred to as the output of [CLS] token (short for “classification”) which is inserted at the beginning of the sentence to output a vector used for classification. Furthermore, *h*_analyst_ is obtained by inputting *x*_analyst_ to MLP consisting of three layers as


(5) $$\begin{array}{l} u=\text{ReLU}\left(\text{LayerNorm}\left(W_{\text{analyst}}\cdot x_{\text{analyst}}+b_{\text{analyst}}\right)\right), \end{array}$$

(6) $$\begin{array}{l} h_{\text{analyst}}=\text{LayerNorm}\left(W_{u}\cdot u+b_{u}\right). \end{array}$$

Here, *W*_analyst_, *W*_*u*_ are weight matrices; *b*_analyst_, *b*_*u*_ are bias vectors; LayerNorm(·) is layer normalization (Ba et al., 2016), which is represented using the input vector *x* as


(7) $$\begin{array}{l} \text{LayerNorm}\left(x\right)=\frac{x-\bar{x}}{\sqrt{\text{Var}\left[x\right]+\epsilon }}\cdot \gamma +\beta . \end{array}$$

Here, β, γ are the learnable parameters; ϵ is a constant; Var[*x*] is the variance of *x*; $\bar{x}$ is the average of *x*. We adopt ReLU^2^ and Layer Normalization in this research since it is empirically known that these contribute to the stability of training. The vector *h* is obtained by concatenating *h*_text_ and *h*_analyst_ as


(8) $$\begin{array}{l} h=\left[\begin{array}{c} h_{\text{text}}\\ h_{\text{analyst}} \end{array}\right]. \end{array}$$

Multilayer perceptron is used to the obtained vector *h*, and the output *y* is obtained as


(9) $$\begin{array}{ll} v & =\text{ReLU}\left(\text{LayerNorm}\left(W_{h}\cdot h+b_{h}\right)\right) \end{array}$$

(10) $$\begin{array}{ll} y & =\text{softmax}\left(W_{v}\cdot v+b_{v}\right). \end{array}$$

Here, *W*_*h*_, *W*_*v*_ are weight matrices, and *b*_*h*_, *b*_*v*_ are bias vectors.

Figure 2B shows an overview of inputting opinion and non-opinion sentences in an analyst report. Let the opinion and non-opinion sentences be *x*_opinion_ and *x*_non−opinion_, respectively. The opinion and non-opinion sentences are input to the BERT models, BERT_opinion_(·) and BERT_non−opinion_(·), respectively, as given as


(11) $$\begin{array}{ll} h_{\text{opinion}} & =\text{BER}{\text{T}}_{\text{opinion}}\left(x_{\text{opinion}}\right) \end{array}$$

(12) $$\begin{array}{ll} h_{\text{non}-\text{opinion}} & =\text{BER}{\text{T}}_{\text{non}-\text{opinion}}\left(x_{\text{non}-\text{opinion}}\right). \end{array}$$

Here, $h_{\text{opinion}}\in {\mathbb{R}}^{d},h_{\text{non}-\text{opinion}}\in {\mathbb{R}}^{d}$, and *d* is the dimension of the hidden layer of BERT. *H*∈ℝ^*d*×2^ is defined as the concatenation of *h*_opinion_ and *h*_non−opinion_ as


(13) $$\begin{array}{l} H=\left[h_{\text{opinion}};h_{\text{non}-\text{opinion}}\right]. \end{array}$$

The weight of the attention mechanism α_*i*_(*i* = 1, 2) is obtained as


(14) $$\begin{array}{l} \alpha_{i}=\frac{e^{s_{i}}}{\overset{2}{\underset{j=1}{\sum }}e^{s_{j}}}, \end{array}$$

where


(15) $$\begin{array}{l} s=\text{tanh}\left(w_{s}\cdot H+b_{s}\right), \end{array}$$

Here, $w_{s}\in {\mathbb{R}}^{d}$ is a weight vector, and $b_{s}\in {\mathbb{R}}^{2}$ is a bias vector. *h* is weighted by the weight α_*i*_, and *h*_text_ is calculated as


(16) $$\begin{array}{l} h_{\text{text}}=\alpha_{1}\cdot h_{\text{opinion}}+\alpha_{2}\cdot h_{\text{non}-\text{opinion}}. \end{array}$$

Here, *h*_text_ and *h*_analyst_ are concatenated according to Equation (8). The output *y* is obtained from Equations (9) and (10).

## 5. Experiments and Results

### 5.1. Opinion Sentence Extraction

The analyst report sentences described in Section 3.1 are used as the input data. The probabilities of the opinion sentences and the non-opinion sentence are output. Among the analyst report sentences, 68%, 12%, and 20% are used as the training, validation, and test data, respectively. We experiment with our method and comparative method of LSTM and MLP (Suzuki et al., 2020). We fix batch size with 32 in our method and 64 in the comparative method. We search hyperparameters from 1 to 60 for the number of epochs and from 10^−6^ to 10^−4^ for the learning rate. We used PyTorch^3^ (version 1.8.1) for implementation, optuna^4^ (version 2.0.0) for hyperparameter optimization, cross-entropy as the loss function, and Adam as the optimization algorithm. These settings are the same in the experiments of net income and excess return.

The result is shown in Table 2. The results show that the proposed model outperforms the comparison methods. We use the proposed model to distinguish between opinion and non-opinion sentences for each sentence in the analyst report set used as input in Section 5.2.

**Table 2:** Result of opinion sentence extraction.

**Model**	**Macro-F1**	**Recall**	**Precision**
Our model	0.811	0.848	0.733
LSTM	0.785	0.767	0.729

### 5.2. Estimating Net Income and Excess Return

We perform the forecast of the magnitude of the increase rate of the analyst's estimated net income and the positive or negative prediction of the excess return using the analyst report set and the analyst profile. There are four types of input sentences in the analyst reports such as full analyst report, opinion sentences only, non-opinion sentences only, and opinion and non-opinion sentences separately. For each input, we conduct experiments with and without the analyst profile. In the experiment without the analyst profile, *h* = *h*_text_ is input in the subsequent MLP instead of the concatenation of *h*_text_ and *h*_analyst_ in Equation (8). As the comparison method, the combination of LSTM and MLP is used as in Section 5.1. In the comparison method of the combination of LSTM and MLP using the analyst profile, the output of the attention mechanism and MLP are concatenated and input to the subsequent MLP. For each input data, 68%, 12%, and 20% are used as the training, validation, and test data, respectively. Tables 3, 4 present the result of forecasting the movement of net income and excess return, respectively. We perform Welch's t-test of confidence of the model in the correct labels of the test data. * in the tables indicates that there is a significant difference at 1% between the model with and without the analyst profile. † in the tables shows that there is a significant difference of 1% compared with this method using the full text using the analyst profile.

**Table 3:** Result of forecasting the magnitude of the change rate of the analyst's estimated net income. The evaluation method is Macro-F1.

**Model**	**Full text**	**Opinion sentences**	**Non-opinion sentences**	**Opinion and Non-opinion sentences**
Our method w/ analyst profile	**0.647** ^*^	0.642^*†^	0.607^*†^	0.640^†^
Our method w/o analyst profile	0.643^†^	0.634^†^	0.595^†^	0.642^†^
LSTM w/ analyst profile	0.636^†^	0.621^*†^	0.593^*†^	0.631^*†^
LSTM w/o analyst profile	0.628^†^	0.615^†^	0.573^†^	0.620^†^

*Significant at 1% compared to the method without the analyst profile.

*Significant at 1% compared to our method with the analyst profile using the full texts. Top score is in bold*.

**Table 4:** Result of forecasting the movement of excess return. The evaluation method is Macro-F1.

**Model**	**Full text**	**Opinion sentences**	**Non-opinion sentences**	**Opinion and Non-opinion sentences**
Our method w/ analyst profile	**0.567** ^*^	0.540^†^	0.553^*†^	0.555^*†^
Our method w/o analyst profile	0.563^†^	0.531^†^	0.537^†^	0.549^†^
LSTM w/ analyst profile	0.559^†^	0.553^*†^	0.556^†^	0.557^*†^
LSTM w/o analyst profile	0.544^†^	0.537^†^	0.537^†^	0.545^†^

*Significant at 1% compared to the method without an analyst profile.

*Significant at 1% compared to our method with the analyst profile using the full texts. Top score is in bold*.

## 6. Discussion

In both Tables 3, 4, our method of the full text input using the analyst profile achieves the highest F1. It achieves a higher F1 than our method of the full text input without the analyst profile. This seems to be effective to add the analyst profile. It is thought that the analyst profile reflected the analyst's experience and popularity and contributes to the improvement of accuracy. In this experiment, it is not effective to divide into opinion sentences and non-opinion sentences.

In the net income forecast, the opinion sentences are more effective than non-opinion sentences, whereas, in the excess return forecast, the non-opinion sentences are more effective than the opinion sentences. This reason would be that the analyst's opinion is strongly reflected in the target value of the net income forecast because it is set by the analyst. On the other hand, in the excess return forecast, non-opinion sentences that state facts may be more effective than opinion sentences. In both experiments, the method with the full texts achieves a higher F1 than the method with the opinion sentences and non-opinion sentences. It is considered that the loss of the order (context) of the input sentences is a disadvantage.

We conduct an additional experiment using the analyst profile for each securities company. Tables 5, 6 present the result of forecasting net income and excess return by a securities company, respectively. In many cases, the full-text input achieves a higher F1 than the other input. However, depending on the securities company, the input method other than the full-text achieves higher F1, such as the opinion and non-opinion sentences input in the securities company C in Tables 5, 6.

**Table 5:** Result of forecasting the magnitude of the change rate of the analyst's estimated net income by securities company using our method with the analyst profile. The evaluation method is Macro-F1.

**Securities company**	**Full text**	**Opinion sentences**	**Non-opinion sentences**	**Opinion and Non-opinion sentences**
A	**0.647**	0.640	0.606	0.637
B	**0.617**	0.595	0.587	0.606
C	0.654	0.649	0.580	**0.675**
D	0.630	0.630	0.581	**0.639**
E	**0.692**	0.653	0.657	0.674

*Top scores per securities company are in bold*.

**Table 6:** Result of forecasting excess return by securities company with our method using the analyst profile. The evaluation method is Macro-F1.

**Securities company**	**Full text**	**Opinion sentences**	**Non-opinion sentences**	**Opinion and Non-opinion sentences**
A	**0.589**	0.552	0.563	0.573
B	0.544	**0.545**	0.540	**0.545**
C	0.543	0.524	0.529	**0.550**
D	**0.540**	0.529	0.522	0.539
E	**0.557**	0.549	0.555	0.553

*Top scores per securities company are in bold*.

## 7. Conclusion

In this study, we apply the analyst report texts and profiles to the model combining BERT and MLP and forecast the magnitude of the change rate of the analyst's estimated net income and the positive/negative excess return. We first divide the analyst reports into opinion and non-opinion sentences, assuming that the analyst's opinion is effective in forecasting change in the estimated net income. Consequently, we achieve F1 exceeding 0.8 in distinguishing between opinion and non-opinion sentences.

Next, we forecast the magnitude of the change rate of the analyst's estimated net income and positive or negative excess return using the opinion and non-opinion sentences extracted from the analyst reports and the analyst profile. For each forecasting, the full-text method using the analyst profile achieves the highest F1. Although there is no effect of separating opinion and non-opinion sentences, the analyst reports texts and profiles are effective.

Since we focused on the effectiveness of the analyst profile in this research, we conducted experiments within a fixed period. However, changes in results when the period is changed may not be negligible. Therefore, the experiment with the different periods is a future task. It is also conceivable to use analyst reports and analyst profiles before the time of forecasting. Using the information from analyst reports and profiles written about the same analyst and stock brand in the past could improve the prediction accuracy. Furthermore, weighting the analyst reports and profiles to obtain reliability instead of weighting equally might be useful for portfolio construction. This research showed an example of effective utilization of information other than the text information of the analyst report. In the future, we will use it with other numerical information and text information, such as tick data.

## Data Availability Statement

The data analyzed in this study is subject to the following licenses/restrictions: Data used in this study is limited to authorized researchers. Requests to access these datasets should be directed to MS, b2019msuzuki@socsim.org.

## Author Contributions

MS organized and executed the experiments and wrote most of the articles. HS contributed to the concept and helped to write the article. KI is a supervisor and administrated the project. YI curated the data and reviewed the article. All authors contributed to the article and approved the submitted version.

## Conflict of Interest

YI is employed by Nikko Asset Management Co., Ltd. The remaining authors declare that the research was conducted in the absence of any commercial or financial relationships that could be construed as a potential conflict of interest.

## Publisher's Note

All claims expressed in this article are solely those of the authors and do not necessarily represent those of their affiliated organizations, or those of the publisher, the editors and the reviewers. Any product that may be evaluated in this article, or claim that may be made by its manufacturer, is not guaranteed or endorsed by the publisher.

## Footnotes

^1^ https://github.com/cl-tohoku/bert-japanese/tree/v1.0

^2^We tried other activation functions such as tanh. However, ReLU had higher performance and stability in training.

^3^ https://github.com/pytorch/pytorch

^4^ https://github.com/optuna/optuna
